# An Audit of Iron Deficiency in Hospitalised Heart Failure Patients: A Commonly Neglected Comorbidity

**DOI:** 10.7759/cureus.41515

**Published:** 2023-07-07

**Authors:** Khalid AlAayedi

**Affiliations:** 1 Acute Medicine, St. Vincent's University Hospital, Dublin, IRL

**Keywords:** iron therapy, ferric carboxymaltose, length of hospitalization, extended hospitalization stay, heart failure prognosis, chronic heart failure, iron-deficiency, decompensated heart failure, heart failure with reduced ejection fraction

## Abstract

Introduction

Iron deficiency (ID) is a common comorbidity in patients with heart failure (HF) and can significantly impact morbidity and mortality, regardless of the presence of anaemia.

Aim

This audit aimed to assess the current practice in diagnosing and assessing iron deficiency (ID) in hospitalised patients with heart failure and reduced ejection fraction (HFrEF). The primary goal was to determine the prevalence of ID in HF patients and the frequency of iron testing in those patients. Additionally, the secondary aims included evaluating the presence of anaemia, the length of hospital stay, and the adequacy of appropriate management for iron deficiency in this patient population.

Methods

A retrospective audit was conducted, reviewing data from patients admitted to St. Vincent University Hospital over a period of 4 months.

Results

Out of the 111 patients audited, only 74% (82) had their iron status checked, and among those tested, 63% (52) met the criteria for iron deficiency according to the European Society of Cardiology (ESC). Additionally, 54% (28) of iron-deficient patients were also anaemic. Iron replacement was administered to 34 out of the 52 patients diagnosed with iron deficiency, accounting for 65% of the identified cases. The average duration of hospital stay for patients with iron deficiency was 13.8 days, while those without iron deficiency had a shorter mean length of stay of 11.2 days. However, it is important to note that the presence of co-morbidities and other confounding factors might have influenced these results.

Conclusion

Despite guideline recommendations, iron deficiency remains under-recognised and undertreated in clinical practice among heart failure patients. There is a crucial need for increased awareness, education, and practical guidance to improve the screening, diagnosis, and management of iron deficiency in hospitalised heart failure patients.

## Introduction

Iron deficiency (ID) is a common comorbidity in patients with heart failure (HF), which in the presence or absence of anaemia can have a significant impact on morbidity and mortality [1,2]. ID can exacerbate HF symptoms and is associated with poorer quality of life, poorer exercise capacity, poorer prognosis, and increased hospitalisation [1,2]. According to the findings of an international pooled analysis involving patients from five cohorts in Poland, Spain, and the Netherlands, iron deficiency has emerged as a significant problem in heart failure, affecting up to 50% of all patients in Europe [3,4]. The 2021 European Society of Cardiology (ESC) guidelines on heart failure have been recently updated, recognising the significance of iron deficiency (ID) in patients with HF and reduced ejection fraction [4]. The guidelines offer detailed instructions on how to diagnose and treat ID, defining it as a serum ferritin level of less than 100 ng/mL or a transferrin saturation (TSAT) of less than 20% when ferritin levels are between 100-300 ng/mL [5]. Consequently, the current guidelines propose that the diagnosis and treatment of ID should form an important component in the assessment of heart failure patients. However, iron deficiency remains under-recognised and undertreated in clinical practice, likely due in part to a lack of practical guidance for clinicians that can be easily followed. The primary aim of this audit was to evaluate the current practice in the diagnosis and assessment of ID in patients admitted to our hospital with a diagnosis of heart failure.

## Materials and methods

The main aim of this audit was to assess the current methods used to diagnose and evaluate iron deficiency (ID) in hospitalised patients with heart failure and reduced ejection fraction (HFrEF). The primary objective was to ascertain the prevalence of ID in HF patients and the frequency of iron testing. Additionally, the secondary goals involved evaluating the occurrence of anaemia, the length of hospital stays, and the frequency of appropriate management for iron deficiency in this particular patient group.

This study aimed to conduct a retrospective audit of patients admitted to St. Vincent University Hospital between July 2022 and October 2022 with a diagnosis of heart failure. Patients aged 18 and above with heart failure, acutely decompensated heart failure, New York Heart Association (NYHA) functional class II-III, and a left ventricular ejection fraction (LVEF) of 40% or lower were included in the study. Excluded from the study were patients with a history of acquired iron overload, known active infection, clinically significant bleeding, active malignancy, chronic liver disease, anaemia unrelated to iron deficiency, and those undergoing immunosuppressive therapy or renal dialysis. The electronic records, discharge letters, and charts of the identified patients were thoroughly examined to collect relevant data for analysis.

The primary objective was to assess each patient's ejection fraction and iron status and determine whether they met the European Society of Cardiology (ESC) criteria for iron deficiency (ID). To accomplish this, the following variables were considered: serum ferritin and transferrin saturation. Haemoglobin levels were also checked to assess how many of the ID patients were also anaemic. To determine if the patients met the ESC criteria for ID, the established thresholds were used, namely ferritin < 100 μg/L or ferritin levels between 100 and 300 μg/L with a transferrin saturation (TSAT) < 20%. The secondary aim was to assess the length of hospital stays, the prevalence of anaemia in patients with iron deficiency, and the frequency of iron replacement.

## Results

**Figure 1:**
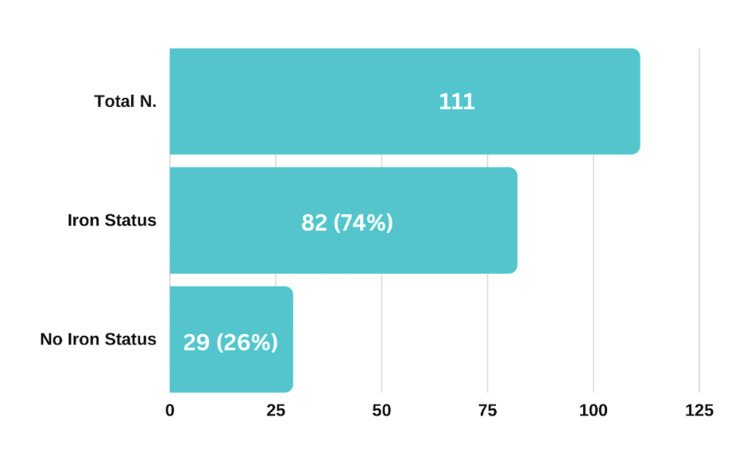
Iron assessment in heart failure patients from July-October2022

**Figure 2:**
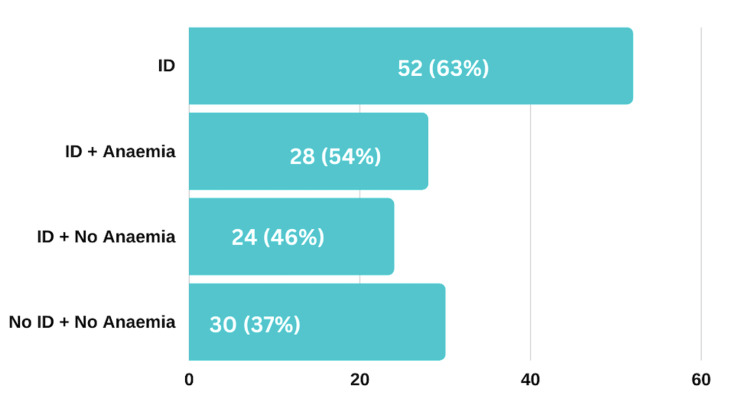
Prevalence of iron deficiency (ID) and anaemia in heart failure

During the audit period, a total of 111 patients were admitted with heart failure, with 82 of them (74%) undergoing an assessment of their iron profile (Figure 1). Among the patients tested, approximately 63% (52) met the European Society of Cardiology (ESC) criteria for iron deficiency (Figure 2). Additionally, out of the 52 patients diagnosed with iron deficiency, 54% (28) were found to have coexisting anaemia. Regarding iron replacement therapy, 65% (34) of the patients diagnosed with iron deficiency received iron replacement after assessment, while the remaining 35% (18) did not (Figure 4).

**Figure 3:**
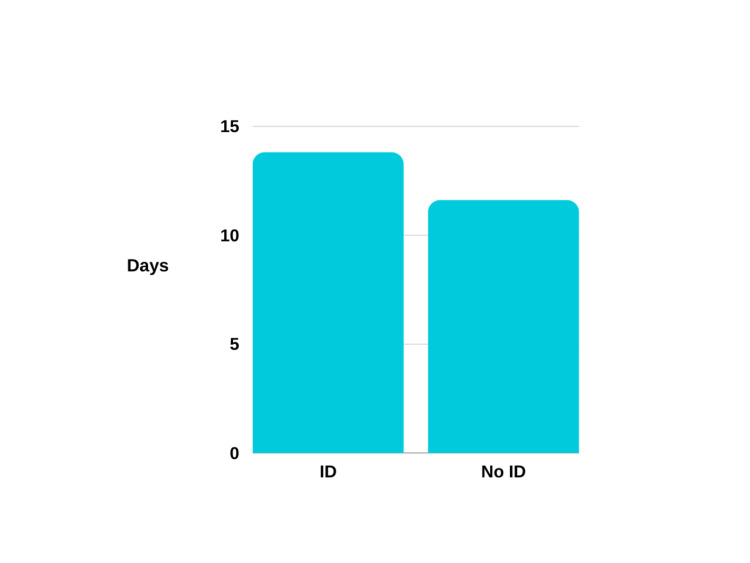
Average length of stay in hospital ID: iron deficiency

**Figure 4:**
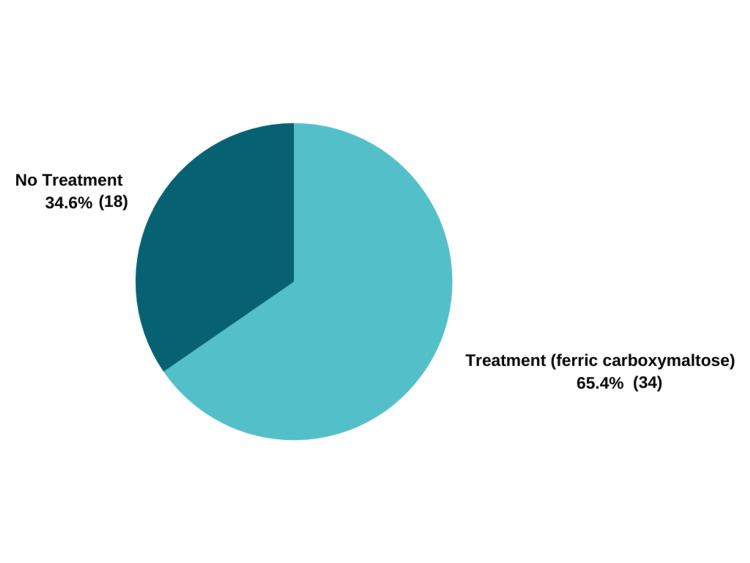
Number of iron deficiency patients who received IV ferric carboxymaltose

Furthermore, the audited heart failure patients with iron deficiency experienced a longer duration of hospitalisation compared to those without iron deficiency. The mean length of hospital stay for patients with iron deficiency was 13.8 days, whereas patients without iron deficiency had a mean length of stay of 11.2 days. However, it is important to note that drawing definitive conclusions from a limited sample size is challenging, and these findings may have been influenced by confounding factors, particularly when considering patients' co-morbidities.

Among the total patients, 16 (14.4%) were admitted under the cardiology team, and all of them had their iron profiles assessed. It should be noted that these patients were more likely to have advanced heart failure and experience complicated hospital admissions compared to the remaining patients admitted under general medical teams.

## Discussion

The results of this audit indicate that only 74% of patients with heart failure had their iron status checked during their hospital admission, and among those who were tested, a majority were iron deficient as per ESC criteria. The prevalence of ID in this audit is consistent with previously reported studies. In patients experiencing acute decompensated heart failure, ID was found in 54% of those with heart failure with reduced ejection fraction (HFrEF) and 56% of those with heart failure with preserved ejection fraction (HFpEF). However, only in the HFrEF group was iron deficiency independently linked with extended hospital stays [6]. However, the prevalence of ID tends to be higher in patients with concomitant anaemia.

It is important to acknowledge that the definition of ID in studies on heart failure (HF) can vary. The widely accepted definition, also endorsed by the European Society of Cardiology (ESC), defines ID as a ferritin level below 100 μg/L or ferritin levels between 100 and 300 μg/L with a transferrin saturation (TSAT) below 20% [5]. However, it should be noted that ferritin, despite being a commonly used biomarker for detecting iron deficiency, can be significantly influenced by factors like inflammation, infection, and malignancy. In an inflammatory state like heart failure, serum ferritin levels may be falsely elevated and may not accurately reflect iron availability [5,6]. Therefore, the ESC definition of ID (also called the FAIR-HF definition) is limited by heavily relying on ferritin levels. For example, Grote Beverborg et al. conducted a study with the objective of establishing and validating a biomarker-based definition of ID in heart failure (HF) by comparing it to the gold standard of bone marrow aspiration with iron staining, which serves as the gold standard [7,8]. The results showed that a transferrin saturation (TSAT) level of ≤19.8% or a serum iron level of ≤13 µmol/L exhibited the best performance in accurately identifying patients with ID and identifying HF patients at the highest risk of mortality [7]. These findings support the existing practice of using a TSAT cutoff of <20% to diagnose ID in HF patients while raising questions about the diagnostic value of ferritin [5].

Iron deficiency (ID) is strongly correlated with a decline in quality of life (QOL), reduced exercise performance, increased disease severity, and poorer prognosis [1,2,6]. In patients with acute heart failure and iron deficiency, the use of intravenous ferric carboxymaltose has shown a potential decrease in the number of hospitalisations due to heart failure and cardiovascular deaths [9,10,11]. This positive impact on symptoms has been demonstrated in both the CONFIRM-HF study conducted in 2014 [9,10] and the AFFIRM-AHF trial conducted in 2020 [10,11]. A study revealed that the administration of intravenous iron therapy led to a considerable decrease in NT-proBNP (N-terminal pro-B-type natriuretic peptide) levels and inflammatory status among anaemic patients with congestive heart failure and moderate chronic renal failure [12]. This positive outcome was associated with improvements in left ventricular ejection fraction (LVEF), NYHA functional class, exercise capacity, renal function, and overall quality of life.

Additionally, this audit shows that a significant proportion of patients with ID were also anaemic. Anaemia in patients with HF is associated with worse outcomes, including higher hospitalisation rates and increased mortality [13]. The aetiology of anaemia in heart failure is multifactorial, with absolute and functional iron deficiency, decreased erythropoietin levels and erythropoietin resistance, inflammatory state and heart failure medications being the important causative factors [14]. Heart failure can lead to anaemia via a large number of mechanisms, and anaemia, in turn, can lead to an increased cardiac workload and possible further deterioration of cardiac function and prognosis [15]. Therefore, the presence of anaemia in ID patients prompts further investigations to exclude other pathologies.

The fact that nearly two-thirds of the tested patients had ID highlights the need for a more routine assessment of iron status in patients with heart failure. Despite the guidelines advocating for its recognition and treatment, iron deficiency remains insufficiently identified and managed in real-world clinical practice. As a result, numerous patients are being deprived of a therapy that could potentially improve both their cardiovascular function and quality of life. This situation may be attributed, at least in part, to the absence of practical guidance regarding the screening, diagnosis, and treatment of iron deficiency. Therefore, changes need to be implemented to increase awareness and education, aiming to equip medical doctors with the necessary knowledge and skills to effectively identify and address iron deficiency in individuals admitted with heart failure.

Interestingly, the audit results highlighted that heart failure patients admitted under the cardiology team were more likely to have their iron status assessed. This finding demonstrates the importance of a multidisciplinary approach in managing heart failure, as the cardiology team's involvement likely facilitated the appropriate evaluation of iron status in these patients. Collaborative efforts between cardiology and other specialities can enhance the overall care and management of heart failure patients, ensuring that potential co-morbidities such as iron deficiency are adequately addressed.

Limitations of this study include its retrospective nature, reliance on electronic records, and potential for missing or incomplete data. Nonetheless, this audit provides valuable insights into the iron status of patients with heart failure and their adherence to ESC criteria for ID.

## Conclusions

The findings of this audit underscore the importance of assessing iron status in patients with heart failure, as a significant percentage of patients demonstrated iron deficiency (ID) according to ESC criteria. The prevalence of ID aligns with previous studies and is particularly notable in heart failure patients with reduced ejection fraction (HFrEF) and those with concomitant anaemia. However, despite guideline recommendations, iron deficiency remains underdiagnosed and undertreated in clinical practice. This emphasizes the need for increased awareness, education, and practical guidance to ensure effective screening, diagnosis, and management of iron deficiency in hospitalized heart failure patients.
